# The *Aspergillus fumigatus* Dihydroxyacid Dehydratase Ilv3A/IlvC Is Required for Full Virulence

**DOI:** 10.1371/journal.pone.0043559

**Published:** 2012-09-18

**Authors:** Jason D. Oliver, Sarah J. Kaye, Danny Tuckwell, Anna E. Johns, Darel A. Macdonald, Joanne Livermore, Peter A. Warn, Mike Birch, Michael J. Bromley

**Affiliations:** 1 F2G Limited, Manchester, United Kingdom; 2 School of Translational Medicine, The University of Manchester, Manchester, United Kingdom; University of Minnesota, United States of America

## Abstract

Dihydroxyacid dehydratase (DHAD) is a key enzyme in the branched-chain amino acid biosynthetic pathway that exists in a variety of organisms, including fungi, plants and bacteria, but not humans. In this study we identified four putative DHAD genes from the filamentous fungus *Aspergillus fumigatus* by homology to *Saccharomyces cerevisiae* ILV3. Two of these genes, AFUA_2G14210 and AFUA_1G03550, initially designated AfIlv3A and AfIlv3B for this study, clustered in the same group as *S. cerevisiae* ILV3 following phylogenetic analysis. To investigate the functions of these genes, AfIlv3A and AfIlv3B were knocked out in *A. fumigatus*. Deletion of AfIlv3B gave no apparent phenotype whereas the *Δilv3A* strain required supplementation with isoleucine and valine for growth. Thus, AfIlv3A is required for branched-chain amino acid synthesis in *A. fumigatus*. A recombinant AfIlv3A protein derived from AFUA_2G14210 was shown to have DHAD activity in an *in vitro* assay, confirming that AfIlv3A is a DHAD. In addition we show that mutants lacking *AfIlv3A* and ilv3B exhibit reduced levels of virulence in murine infection models, emphasising the importance of branched-chain amino acid biosynthesis in fungal infections, and hence the potential of targeting this pathway with antifungal agents. Here we propose that AfIlv3A/AFUA_2G2410 be named *ilvC*.

## Introduction

*Aspergillus fumigatus* is a filamentous fungus and an opportunistic human pathogen, which primarily affects immunocompromised patients. The numbers of patients with invasive fungal infections has risen dramatically over the last 20 years [1], [2]. A major contribution to the increase in fungal infection is the increasing number of people with impaired immunological defences due to disease or medical intervention, such as, transplant recipients, cancer patients, persons treated with corticosteroids and HIV patients. Existing antifungal therapies have various limitations such as limited spectrum, toxicity, increasing resistance and drug-drug interactions [3]. New antifungals with novel modes of action are required to address the medical need.

Certain fungal biosynthetic pathways provide attractive targets for antifungal research because they are absent in mammals. One such pathway is the branched-chain amino acid biosynthetic pathway, which synthesises isoleucine, leucine and valine in a range of organisms from bacteria to eukaryotes, including fungi and higher plants [4]. This pathway is the target of several different herbicides that inhibit the first common enzyme in the pathway, acetolactate (also known as acetohydroxyacid) synthase [5], [6]. Results from several studies on different fungi indicate that acetolactate synthase may also be a good target for antifungal therapy. *Saccharomyces cerevisiae ilv2Δ* has greatly decreased ability to grow *in vivo* [7], and virulence is decreased when *ILV2* is disrupted in *Cryptococcus neoformans* [8] and *Candida albicans* [9].

Dihydroxyacid dehydratase (DHAD, EC 4.2.1.9) is two steps downstream of acetolactate synthase in the branched-chain amino acid biosynthetic pathway. DHAD catalyses the dehydration of 2,3-dihydroxy-3-methylvalerate and 2,3-dihydroxyisovalerate to the corresponding ketoacids, 2-keto-3-methylvalerate and 2-ketoisovalerate respectively [10]. This enzyme is present in a variety of archae, bacteria and eukaryotes, including *Methanococcus spp.*, *Escherichia coli*, and *Spinach oleracea* [11]–[15]. In the yeast *S. cerevisiae* the *ILV3* gene codes for DHAD [16], and DHAD activity has been investigated in *Neurospora crassa* [17], but the gene or genes responsible for DHAD activity in *Aspergillus fumigatus* have not been investigated in any detail.

In this study, four ILV3 homologues have been identified in *A. fumigatus*. Phylogenetics, mutagenesis and biochemistry approaches have determined that one of these genes encodes a DHAD that performs a primary role in branched-chain amino acid biosynthesis.

## Results

### A family of ILV3 proteins in *A. fumigatus*

**Figure 1:**
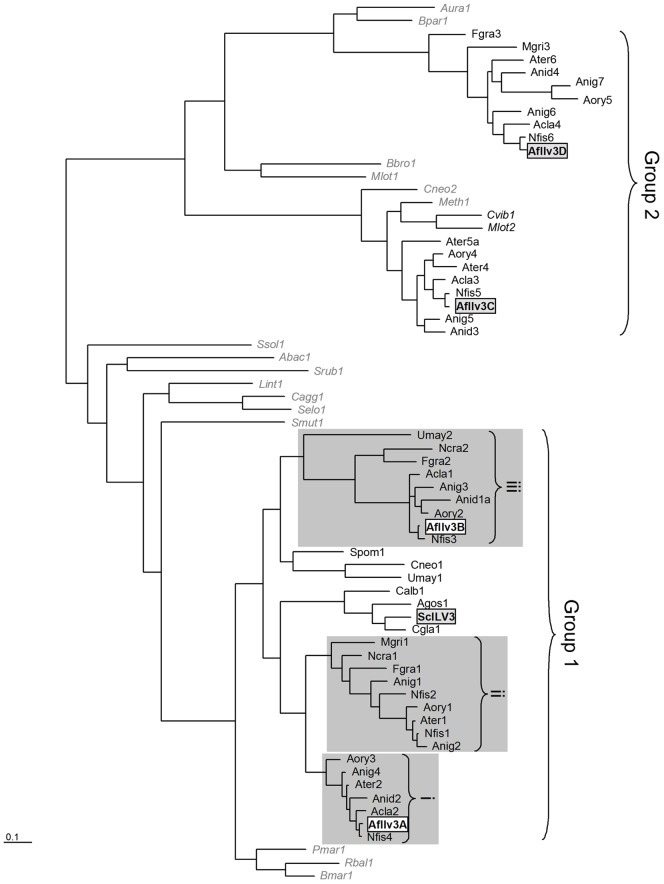
Phylogenetic tree for ILV sequences from fungi, bacteria and archaebacteria. The following sequences were analysed as described in the Experimental Procedures (*S. cerevisiae* ILV3 and *A. fumigatus* Ilv3A, Ilv3B, Ilv3C and Ilv3D are boxed to highlight; bacterial and archaebacterial sequences are in italics): ***FUNGI:*** AfIlv3A, AfIlv3B AfIlv3C AfIlv3D (boxed), *Aspergillus fumigatus;* Agos1 (NP_983287.1), *Ashbya gossypii*; Acla1 (XP_001275730.1), Acla2 (XP_001269800.1), Acla3 (XP_001275913.1), Acla4 (XP_001269430.1), *Aspergillus clavatus*; Anid1 (XP_663950.1), Anid2 (XP_661662.1), Anid3 (XP_680627.1), Anid4, (XP_662742.1), *Aspergillus nidulans*; Anig1 (XP_001400953.1), Anig2 (XP_001398714.1), Anig3 (XP_001390333.1), Anig4 (XP_001398874.1), Anig5 (XP_001397198.1), Anig6 (XP_001392062.1), Anig7 (XP_001394885.1), *Aspergillus niger*; Aory1 (BAE58705.1), Aory2 (BAE60994.1), Aory3 (BAE54877.1), Aory4 (BAE61320.1), Aory5 (BAE65970.1), *Aspergillus oryzae*; Ater1 (XP_001208445.1), Ater2 (XP_001213593.1), Ater3 (XP_001211495.1), Ater4 (XP_001217288.1), Ater5 (XP_001209472.1), *Aspergillus terreus*; Calb1 (CaO19.4040), *Candida albicans*; Cgla1 (XP_445144.1), *Candida glabrata*; Cneo1 (XP_572335.1); Cneo2 (XP_571442.1), *Cryptococcus neoformans*; Fgra1 (XP_382232.1), Fgra2 (XP_382893.1), Fgra3 (XP_386693.1), *Fusarium graminearum*; Mgri1 (XP_359970), Mgri2 (XP_001407130), *Magnaporthe grisea*; Ncra1 (XP_958280.1), Ncra2 (XP_963045.1), *Neurospora crassa*; Nfis1 (XP_001260877.1), Nfis2 (XP_001266525.1), Nfis3 (XP_001262996.1), Nfis4 (XP_001265300.1), Nfis5 (XP_001261093.1), Nfis6 (XP_001264936.1), *Neosartorya fischeri*; Scer1 (ILV3_YEAST), *Saccharomyces cerevisiae*; Spom1 (ILV3_SCHPO), *Schizosaccharomyces pombe*; Umay1 (UM05740.1), Umay2 (UM02980.1), *Ustilago maydis*. ***BACTERIA:***; Abac1 (Q1ILZ0) *Acidobacteria bacterium*; Aura1 (ZP_01227770) *Aurantimonas manganoxydans*; Bbro1 (Q7WKV5) *Bordetella bronchiseptica*; Bmar1 (ZP_01094431) *Blastopirellula marina*; Bpar1 (NP_883684.1) *Bordetella parapertussis*; Cagg1 (AOH553_9CHLR) *Chloroflexus aggregans*; Cvib1 (Q9A8D3) *Caulobacter vibroides*; Lint1 (Q8F219) *Leptospira interrogans*; Meth1 (ZP_01850751.1) *Methylobacterium sp*; Mlot1 (NP_106075.1), Mlot2 (Q986V5), *Mesorhizobium loti*; Pmar1 (ZP_01852636.1) *Planctomyces maris*; Rbal1 (Q7UJ69) *Rhodopirelula baltica*; Selo1 (Q31QL1) *Synechococcus elongatus*; Smut1 (Q8DRT7) *Streptococcus mutans*; Srub1 (Q2SOM3) *Salinabacter ruber*; ***ARCHAEBACTERIA:*** Ssol1 (Q97UB2) *Sulfolobus solfataricus*. The scale bar corresponds to the branch length for an expected number of 0.1 substitutions per site.

In order to identify the *A. fumigatus* ortholog of the *Saccharomyces cerevisiae* ILV3 protein, BLAST searches were carried out, leading to the identification of four *A. fumigatus* proteins. Proteins encoded by the genes AFUA_2G14210, AFUA_1G03550, AFUA_1G07330 and AFUA_2G16300 were 63%, 55%, 31% and 29% identical to *S. cerevisiae* Ilv3p, respectively. For the purposes of clarity they shall be referred to as AfIlv3A (AFUA_2G14210), AfIlv3B (AFUA_1G03550), AfIlv3C (AFUA_1G07330) and AfIlv3D (AFUA_2G16300) in this manuscript. As the relationship of these proteins to ILV3 was not clear, a phylogenetic analysis was undertaken using in addition sequences homologous to ILV3 from other fungi, bacteria and archeabacteria. Phylogenetic analysis was carried out using the more conserved regions of the alignment using Bayesian inference (MrBayes), maximum likelihood (TreePuzzle) and Distance (Protdist) analyses (Figure 1). Two distinct and well-supported clades of fungal ILV3 sequences were seen, given here as groups 1 and 2.

Group 1, which contained *S. cerevisiae* ILV3 also contained *A. fumigatus* AfIlv3A and AfIlv3B. *S. cerevisiae*, *C. albicans*, *Candida glabrata* and *Ashbya. gossypii* only have a single ILV3 sequence, and these clustered together, however in filamentous fungi, three sub-groups were apparent (given as i–iii). The two *A. fumigatus* sequences were in separate groups, and *A. fumigatus* had no sequence in sub-group ii, however some other filamentous fungi had at least one sequence in each sub-group, e.g. *Neosartorya fisheri* and *Aspergillus niger* which have one sequence in sub-group i, two in sub-group ii and one in sub-group iii. Sub-groups i and ii appear to have arisen from a gene duplication after the separation of “yeasts” (Saccharomycotina) and “filamentous fungi” (Pezizomycotina) but it is not clear whether sub-group iii arose before or after this stage. The phylogenetic analysis indicates that, in *A. fumigatus*, AfIlv3A and AfIlv3B are potentially carrying out the same role as the Ilv3 protein of *S. cerevisiae*. In other filamentous fungi with sequences in all three groups, there would be at least three candidates.

Group 2 sequences clustered strongly with α and β proteobacteria *Aurantimonas sp. S185-9A1* (Aura1), *Bordetella parapertussis* (Bpar1), *Bordetella bronchiseptica* (Bbro1), *Mesorhizobium loti* (Mlot1), *Methylobacterium sp. 4–46* (Meth1), and *Caulobacter vibrioides* (Cvib1), suggesting a separate evolutionary lineage from group 1, possibly lateral gene transfer in one case, however, since Basidiomycete sequences were observed, any transfer is likely to have occurred a long time ago. Within group 2, two sub-groups were observed, with *A. fumigatus* sequences present in both groups. Other filamentous fungi also had representatives in both groups, and in the case of *A. niger*, two sequences were seen in the same sub-group. Phylogenetic analysis therefore demonstrated that there is a large and non-uniform family of ILV3 sequences in filamentous fungi, quite unlike the situation seen in *S. cerevisiae*, and that in the case of *A. fumigatus*, there are two candidate ILV3 proteins, AfIlv3A and AfIlv3B.

### AfIlv3A is predicted to be a mitochondrial protein

**Figure 2:**
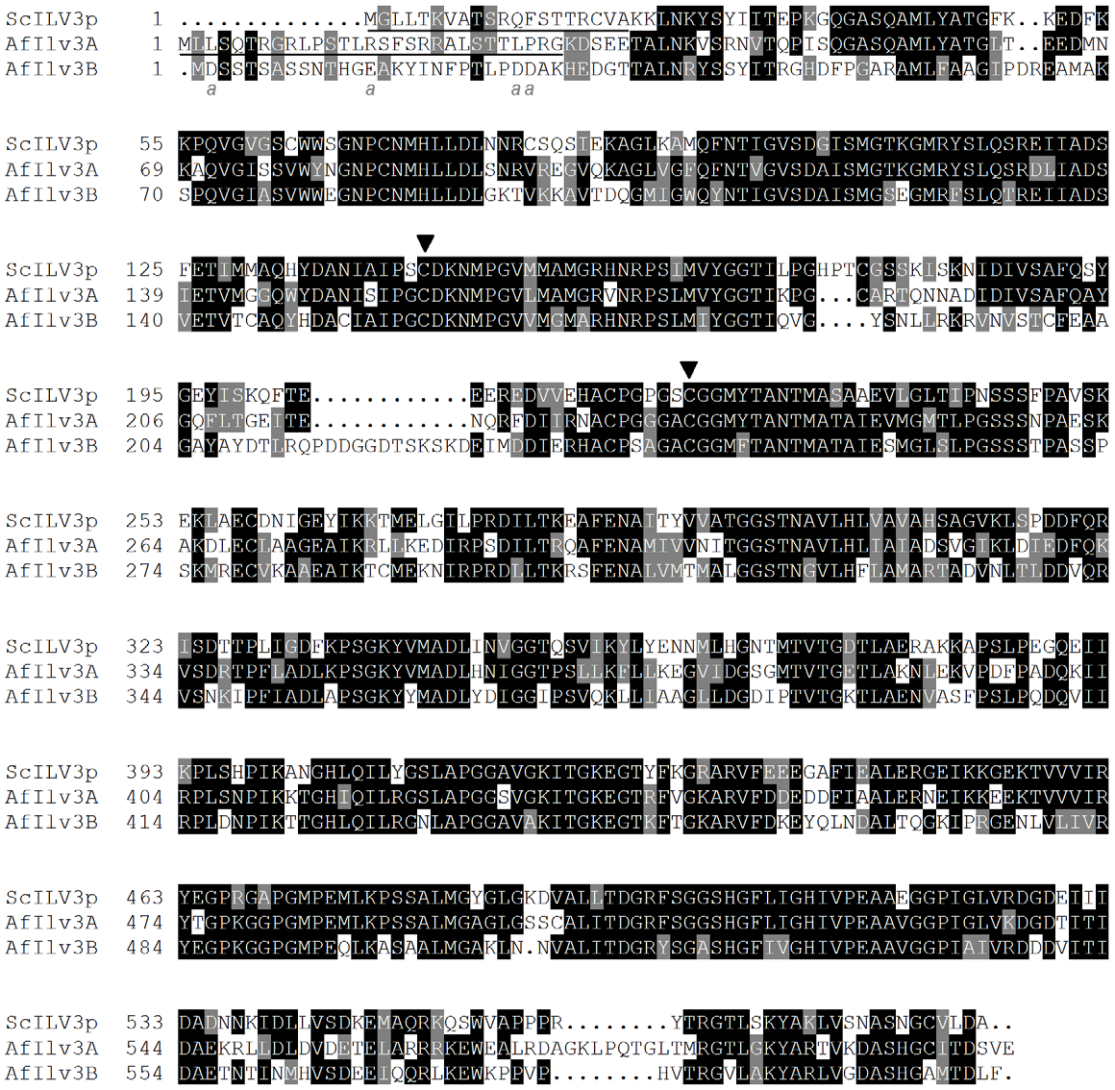
Alignment of *S. cerevisiae* Ilv3p with *A. fumigatus* Ilv3A and *A. fumigatus* Ilv3B. The protein sequences for *S. cerevisiae* Ilv3p (ScIlv3p) and *A. fumigatus* AfIlv3A (product of gene AFUA_2G14210) and AfIlv3B (product of gene AFUA_1G03550) were aligned with ClustalW. Black boxes indicate identical residues and grey boxes indicate similar residues (added by BOXSHADE 3.21). The predicted mitochondrial targeting sequences of ScILV3 and AfIlv3A are underlined. Acidic residues at the N-terminus of AfIlv3B (not usually found in mitochondrial targeting sequences) are indicated by ‘*a*’. Arrowheads indicate cysteine residues conserved between these fungal sequences and DHAD sequences from *E. coli* and *L. lactis* that may be involved in iron-sulphur centres [16].

The DHAD step of branched-chain amino acid biosynthesis is thought to occur in mitochondria in plants and fungi [4], [18]. Ilv3p of *S. cerevisiae* is predicted to have an N-terminal mitochondrial targeting sequence rich in positively charged and hydroxylated residues [16]. To investigate whether any of the *A. fumigatus* Ilv3 proteins are likely to be imported into the mitochondria their predicted amino acid sequences were analysed. For AfIlv3A and AfIlv3B information about the transcriptional start site was gained by 5′ RACE. The RACE data confirmed our prediction that AfIlv3B has an extra exon at the 5′ end compared to that predicted on CADRE for gene AFUA_1G03550. An alignment of the protein sequences of AfIlv3A and AfIlv3B to *S. cerevisiae* Ilv3p is shown in Figure 2.

Three programs were used to determine the likely presence of a mitochondrial targeting sequence: TargetP, MitoProt and Predotar. Of the four *A. fumigatus* ILV3 homologs, only AfIlv3A is predicted to be directed to the mitochondria (Table 1). All three prediction programs indicate that *S. cerevisiae* Ilv3p and AfIlv3A are imported into mitochondria. These data suggests that AfIlv3A is the only Ilv3-like protein from *A. fumigatus* that is located in the mitochondria and is therefore the most likely candidate to function in the branched-chain amino acid synthesis pathway.

**Table 1:** Predictions of mitochondrial import for Ilv3 proteins. The Ilv3-like protein sequences indicated were analysed using TargetP.

	% identity to ScIlv3p	TargetPv1.1	MitoProtV1.101	PredotarV1.03
S. cerevisiae Ilv3pYJR016C	100	0.61	0.946	0.74
AfIlv3AAFUA_2g14210	63	0.93	0.995	0.64
AfIlv3BAFUA_1g03550	55	0.072	0.048	0
AfIlv3C//AFUA_1g07330	31	0.096	0.018	0
AfIlv3DAFUA_2g16300	29	0.055	0.01	0

*(* http://www.cbs.dtu.dk/services/TargetP/ *), Mitoprot (* http://ihg2.helmholtz-muenchen.de/ihg/mitoprot.html *) and Predotar (* http://urgi.versailles.inra.fr/predotar/predotar.html *) web-based analysis programs.*

### Deletion of AfIlv3A and AfIlv3B in *A. fumigatus*

Disruption of genes of the branched-chain amino acid synthesis pathway leads to auxotrophy for isoleucine and valine in *S. cerevisiae* [19], *N. crassa and C. neoformans* [8]. To investigate whether AfIlv3A or AfIlv3B were important for branched-chain amino acid synthesis in *A. fumigatus* the genes AFUA_2G14210 and AFUA_1G03550 were deleted. A Ku80ΔpyrG^−^ strain derived from CEA10 was used as the host for this work to take advantage of the improved frequency of homologous recombination in isolates deficient in non-homologous end joining [20]. Gene knockout cassettes were generated by PCR fusion of fragments flanking the gene target and an *A. nidulans pyrG* selectable marker [21].

Construction of the double null mutant strain (*Δilv3A*, *Δilv3B*) was achieved by recycling the pyrG selection marker in the *Δilv3A* strain on medium containing 5′-fluoroorotic acid followed by transformation with the ilv3B knockout construct. Precise gene replacement was confirmed in all strains by PCR and Southern blotting (Figure S1).

**Figure 3:**
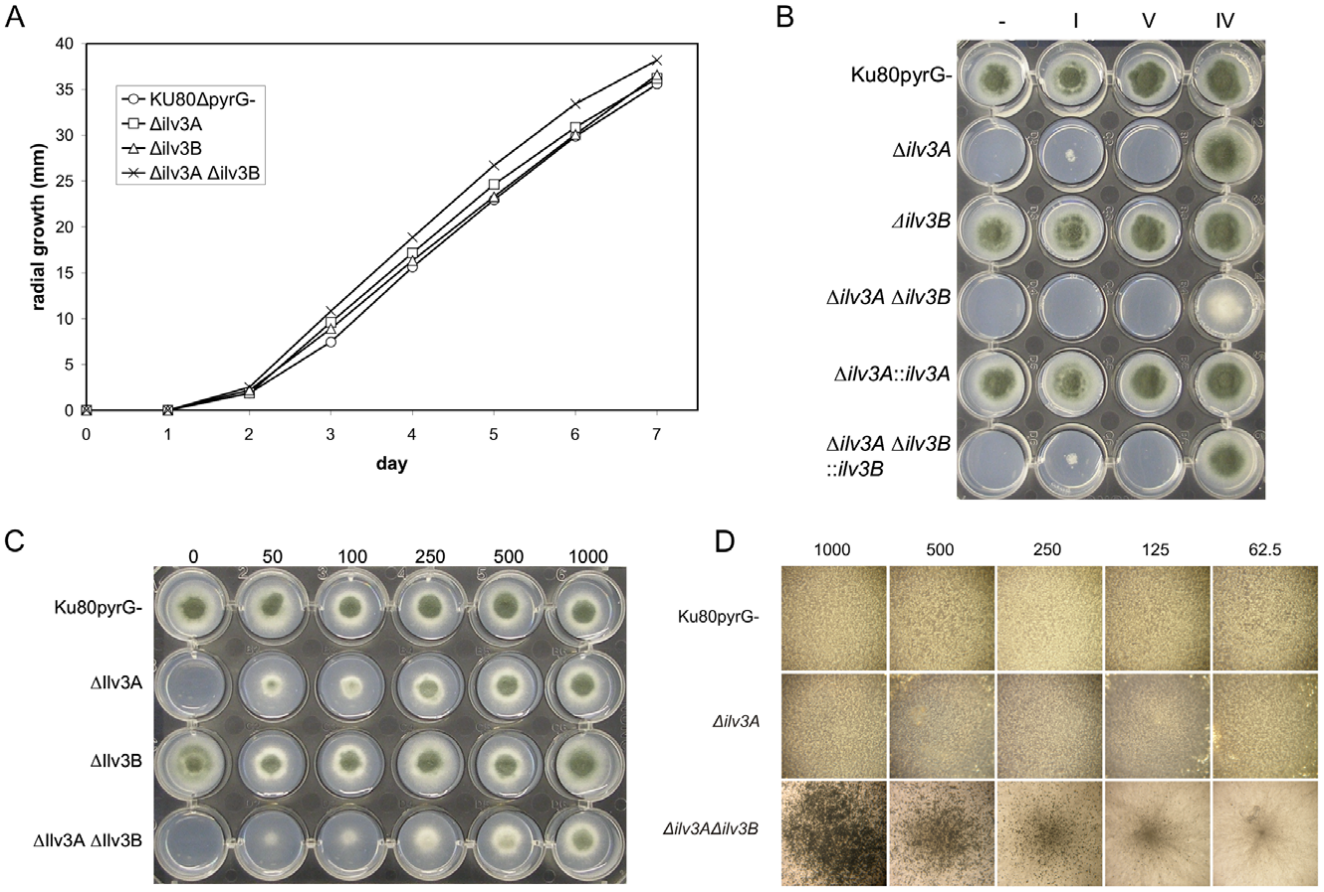
Growth of AfIlv3 knockout strains. **A.** Radial growth rate analysis of the AfIlv3 strains. 200 spores were spotted onto the centre of a minimal agar plate and incubated at 37°C for seven days. Colony size was measured daily. Each datapoint is the mean of 3 measurements. **B.** The indicated strains were inoculated onto minimal agar in the absence (-) or presence of 500 µM of the following amino acids: isoleucine (I); valine (V); isoleucine+valine (IV). The plates were incubated at 37°C for 3 days. **C.** The indicated strains were inoculated onto minimal agar in the presence of 0, 50, 100, 250, 500, 1000 µM isoleucine+leucine+valine. The plates were incubated at 37°C for 3 days. **D.** 200 spores of the indicated strains were spotted into the centre of a minimal agar plate containing 10 mM nitrate as a sole nitrogen source in the presence of 62.5, 125, 250, 500, 1000 µM isoleucine+leucine+valine. Plates were and incubated at 37°C for 3 days. Images were taken at 20× magnification.

Preliminary growth rate analysis, in the presence of supplemental branched-chain amino acids, revealed no obvious differences between the mutant and parental strains (Figure 3A). Furthermore, loss of AfIlv3B had no apparent effect on the ability of *A. fumigatus* to grow in the absence of isoleucine and valine (Figure 3B and 3C). In fact the *Δilv3B* strain had an identical phenotype to the parental strain with regard to branched-chain amino acids. In contrast, the *Δilv3A* strain demonstrated an inability to grow in the absence of isoleucine and grew only minimally in the absence of valine (Figure 3B and 3C). Addition of isoleucine and valine was required to fully complement the phenotype seen for *Δilv3A*. Leucine was not required for growth and interestingly had a negative effect on growth in these experiments. Replacement of AfIlv3A to its native locus led to a parental-type prototrophic phenotype demonstrating that the auxotrophy was due to AfIlv3A disruption and not any unidentified mutation in this strain.

The double knockout strain *Δilv3A Δilv3B* also required both isoleucine and valine for growth. However, there was a clear difference in the appearance of the double null mutant compared to the other strains in the presence of limited amino acids (50–500 µM). Microscopic examination reveals that this isolate was unable to produce asexual spores at low concentrations of branched-chain amino acids (62.5 µM isoleucine, leucine and valine), this phenotype is rescued in a dose dependent manner with further supplementation (Figures 3C and 3D). Therefore AfIlv3B appears to play a role in branched-chain amino acid synthesis in the absence of AfIlv3A.

### Virulence of Ilv3-like knockout strains in murine infection models

**Figure 4:**
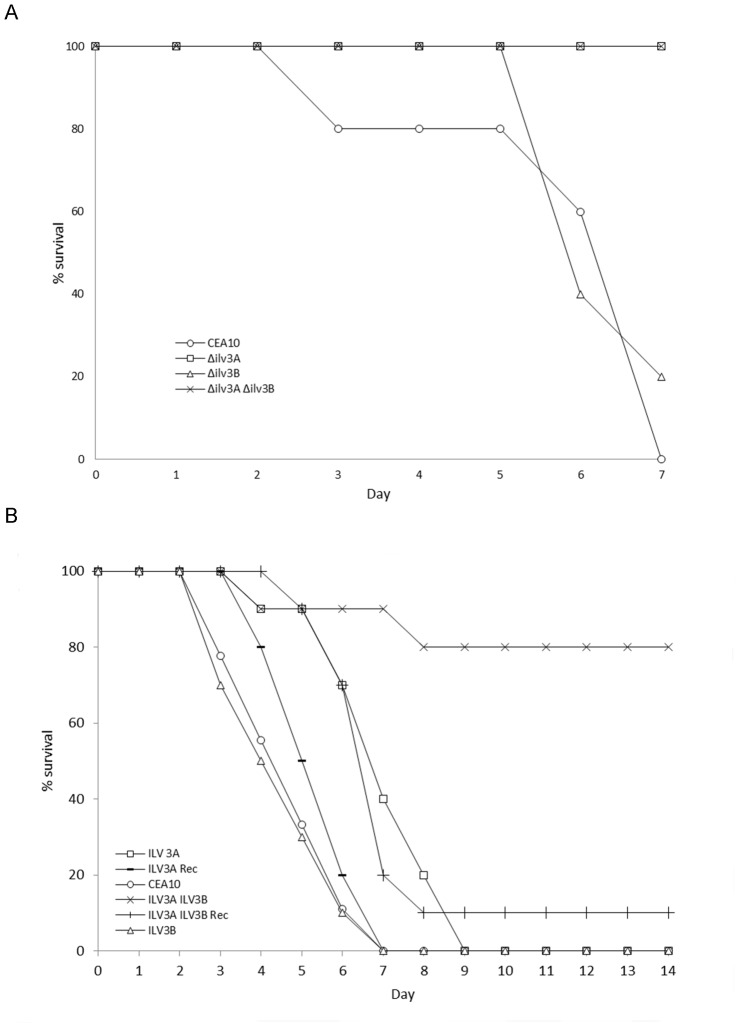
Virulence of AfIlv3A and AfIlv3B knockout strains in murine infection models. **A.** Survival study of mice infected with 2.5×10^5^ spores of the indicated strains inoculated into the tail vein of groups of 5 mice. The number of surviving mice were monitored for 7 days and expressed as a percentage of the starting number. **B.** Survival study of mice infected using the high inoculum inhalational model in groups of 9 or 10. The number of surviving mice were monitored for 14 days and expressed as a percentage of the starting number.

A preliminary study was undertaken to assess the virulence of the knockout strains in male CD1 neutropenic mice infected via the lateral tail vein. This study showed that both *Δilv3A and Δilv3AΔilv3B* resulted in considerably lower kidney tissue burden than either the wild-type CEA10 or *Δilv3B* strains (data not shown). In a survival study using the same model, groups of 5 mice were monitored for 7 days post inoculation (Figure 4A). The *Δilv3B* strain behaved similarly to CEA10 with 1 (20%) and 0 mice surviving respectively, indicating that these strains were both capable of a lethal infection. All mice infected by the *Δilv3A* and the *Δilv3AΔilv3B* strains survived to the end of the study, indicating that the virulence of these strains was severely impaired.

A second model system using a high dose inhalational administration was used to further assess the virulence of the KO strains. Survival groups of 9 or 10 mice were monitored for 14 days following inoculation (Figure 4B). In this study the virulence of *Δilv3A* was significantly different from the wild-type strain (P = 0.0009; Kruskal-Wallis with Conover-Inman pairwise comparison) however all infected mice eventually succumbed to the infection. No difference in virulence was observed between the wild-type and *Δilv3B* strains. Interestingly the *Δilv3AΔilv3B* was more severely attenuated than the *Δilv3A* strain alone (CEA10 vs *Δilv3AΔilv3B* P<0.0001; *Δilv3A* vs *Δilv3AΔilv3B* P = 0.029). As expected the ilv3A reconstituted strain regained full virulence (P = 0.299 vs CEA10) whilst the reintroduction of ilv3B into the *Δilv3AΔilv3B* strain returned virulence to levels similar to that of the *Δilv3A* strain.

Combined these data clearly suggest that *AfIlv3B* is not required for virulence of *A. fumigatus* while loss of *AfIlvA* leads to a reduction in virulence. Loss of both ilv genes leads to a larger decrease in virulence compared to loss of *Afilv3A* alone suggesting that *Afilv3A* and *Afilv3B* have complementary roles..

### Recombinant protein production and dihydroxyacid dehydratase activity

**Figure 5:**
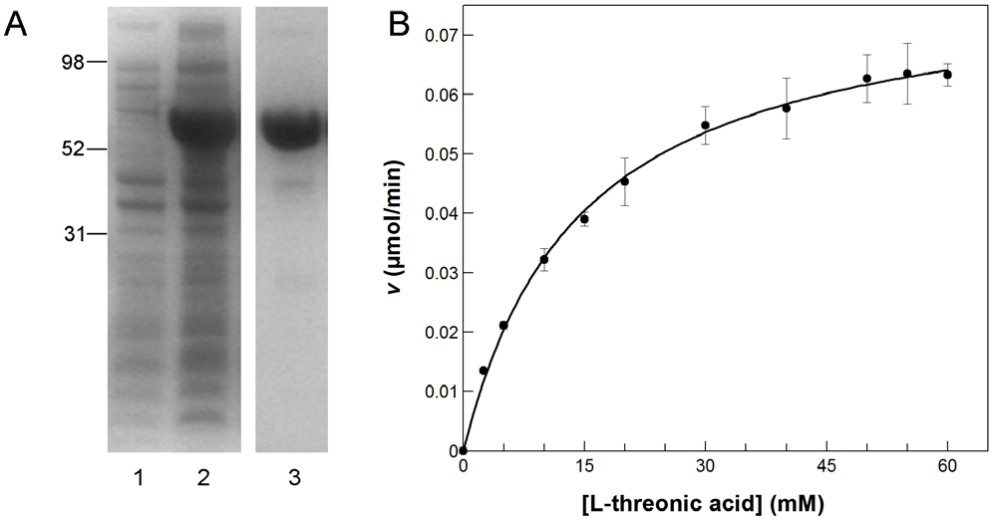
Recombinant AfIlv3A expression, purification and DHAD activity. **A.** Expression of recombinant Ilv3A protein. *E. coli* BL21 DE3 cells were transformed with pET30_Ilv3A, grown until OD600>0.5 and then incubated in the presence and absence of 0.5 mM IPTG for 20 h at 20°C. Ilv3A was purified from the bacterial lysate by immobilized metal ion affinity chromatography (IMAC). Protein fractions were separated by polyacrylamide gel electrophoresis on 4–12% Bis-Tris NuPage gels (Invitrogen) followed by staining with GelCode Blue staining reagent (Pierce). The molecular weights in kilo Daltons of marker proteins are indicated. Lane 1, *E. coli* cell lysate; lane 2, *E. coli* cell lysate following IPTG induction; lane 3, Ilv3A protein purified by IMAC. **B.** Dihydroxyacid dehydratase activity of recombinant Ilv3A. 2 µg Ilv3A was assayed in the presence of 0–60 mM L-threonic acid. Initial reaction velocities were calculated and plotted versus substrate concentration.

A recombinant AfIlv3A obtained from AFUA_2G14210 cDNA and lacking its predicted mitochondrial targeting sequence but with an additional N-terminal His-tag was expressed to high levels in *E. coli*. The purified protein appeared as a single band on SDS-PAGE with apparent molecular weight of approximately 65 kDa (Figure 5A) corresponding well with the calculated molecular weight of 66.4 kDa (including N-terminal His tag). AfIlv3A protein fractions appeared brown in colour, which is likely to be due to the *A. fumigatus* DHAD containing a Fe-S cluster, as reported for DHAD of *E. coli* and *S. oleracea* [12], [22].

AfIlv3A protein was tested for dihydroxyacid dehydratase activity in an assay using the non-natural substrate L-threonate. Other DHAD enzymes have been shown to utilise this alternative substrate, although to lower specific activities than the natural substrate, 2,3-dihydroxyisovalerate [14], [23]. Recombinant AfIlv3A displayed DHAD activity with L-threonate at a specific activity of 18 µmol min^−1^ mg^−1^. Michaelis-Menten type enzyme kinetics were observed and AfIlv3A had a Km for threonate of 10.1 mM (Figure 5B). The DHAD activity of AfIlv3A was inhibited in a dose-dependent manner by 2-hydroxy-3-methylbutyric acid, a known substrate analog inhibitor of DHAD [24], with an IC_50_ of approximately 8 mM.

When similarly overexpressed in *E. coli*, AfIlv3B was found to fractionate into the inclusion bodies in an insoluble form. Upon solubilisation and refolding no DHAD activity was observed (data not shown), possibly due to non-native folding.

## Discussion

Amino acid biosynthesis pathways are major targets of herbicides with 3 main pathways being targeted [6]. Glyphosate targets enolpyruvylshikimate-3-phosphate synthase in the shikimate pathway of aromatic amino acid biosynthesis, glufosinate inhibits glutamine synthetase and several herbicidal chemical families act by inhibiting acetolactate synthase from the branched-chain amino acid biosynthesis pathway.

Can similar pathways be targeted in the fight against microbial infections? A major obstacle to preventing microbial growth by blocking these pathways is that amino acids are readily available in human serum. For the branched-chain amino acids isoleucine, leucine and valine the serum concentration in adults is in the range 51–267 µmol/l [25]. Nevertheless, work in *C. neoformans* and *C. albicans* has suggested that acetolactate synthase, encoded by the *ILV2* gene, has potential to be an antifungal target [8], [9]. Furthermore, sulfometuron methyl, an inhibitor of acetolactate synthase, showed some efficacy against *Mycobacterium tuberculosis* in a mouse model of infection [26].

The dihydroxyacid dehydratase step of branched-chain amino acid biosynthesis has not been previously investigated as a potential antifungal target. A single gene, ILV3 codes for the *S. cerevisiae* dihydroxyacid dehydratase. However, bioinformatic analyses revealed that filamentous fungi appear to have several Ilv3-like proteins (Figure 1). As was recently reported to be the case in *Aspergillus nidulans* [27], four Ilv3 homologs were identified in the human pathogen *Aspergillus fumigatus*. Two of these proteins, AfIlv3A (AFUA_2G14210) and AfIlv3B (AFUA_1G03550), were clustered together phylogenetically with the single Ilv3 proteins present in *S. cerevisiae* and *C. albicans*, and were therefore more likely candidates to be carrying out the same function in *A. fumigatus* as ILV3 does in *S. cerevisiae* (Figure 1).

Several pieces of evidence suggest that AfIlv3A is the primary enzyme that carries out the dihydroxyacid dehydratase step of branched-chain amino acid biosynthesis in *A. fumigatus*. As well as being slightly closer than Ilv3B to *S. cerevisiae* ILV3 on the phylogenetic tree, several programs predict that AfIlv3A, like *S. cerevisiae* ILV3, is imported into the mitochondrial matrix where the bulk of the pathway including the DHAD step are thought to occur, whereas AfIlv3B, AfIlv3C (AFUA_1G07330) and AfIlv3D (AFUA_2G16300) are not predicted to be mitochondrial. The phenotype of the *Δilv3A* strain is consistent with AfIlv3A being required for branched-chain amino acid biosynthesis as it only grows when supplemented with isoleucine and valine. The *Δilv3B* strain is prototrophic for these amino acids. Finally, recombinant AfIlv3A protein has dihydroxyacid dehydratase activity *in vitro*, confirming that this protein has the potential to fulfil this role *in vivo*.

The role of AfIlv3B is unclear, however it may be a cytosolic partner to AfIlv3A. Although the dihydroxyacid dehydratase of *S. cerevisiae* is mitochondrial, it is known that various enzymes of leucine biosynthesis are cytosolic including Leu1p (isopropylmalate isomerase) and Leu2p (Beta-isopropylmalate dehydrogenase) and others are present in both cytosolic and mitochondrial forms including Leu4p (Alpha-isopropylmalate synthase) [4]. Our data precludes a primary role of AfIlv3B in either isoleucine, leucine or valine biosynthesis since the *Δilv3B* mutant grows equally well in the absence of supplementary branched-chain amino acids (Figure 3B). However in the absence of AfIlv3A, AfIlv3B may play some role in branched-chain amino acid metabolism as the double knockout has an altered phenotype compared to *Δilv3A*. The lack of sporulation of the double knockout strain is rescued by high concentrations of isoleucine, leucine and valine indicating that AfIlv3B may contribute to the biosynthesis of these amino acids. Unfortunately we were unable to determine whether AfIlv3B has DHAD activity as the recombinant AfIlv3B expressed in *E. coli* was insoluble.

**Figure 6:**
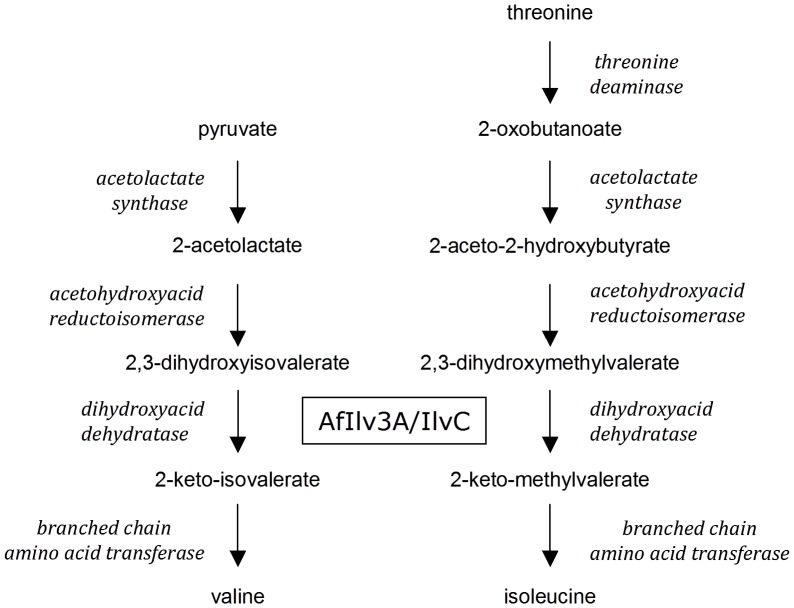
The branched chain amino acid biosynthesis pathway in *A. fumigatus*.

Taken together, these data suggest that AfIlv3A is the enzyme of *A. fumigatus* that converts α,β-dihydroxyacids to α,β-keto acids during the synthesis of isoleucine, leucine and valine in the mitochondria (Figure 6) and in accordance with standard Aspergillus gene nomenclature we propose that the gene AFUA_2G14210 be named *ilvC*. The roles of AfIlv3B (AFUA_1G03550), AfIlv3C (AFUA_1G07330) and AfIlv3D (AFUA_2G16300) are less clear, but the latter two enzymes are evolutionarily distinct from *S. cerevisiae* ILV3 and may have unrelated functions. One pathway linked to DHAD is the pantothenate and coenzyme A biosynthesis pathway. 3-methyl-2-oxobutanoate (keto-isovalerate), the product of DHAD, is an intermediate of pantothenate synthesis. It is possible that AfIlv3B is able to service this pathway in the absence of AfIlv3A*I*IlvC. However, addition of pantothenate to the medium does not appear to alter the phenotype of the *Δilv3A Δilv3B* strain (data not shown). Recent findings linked branched-chain amino acid biosynthesis to the response to hypoxia with several enzymes in the pathway up-regulated during hypoxia [27]. The four DHAD genes were differently regulated in the hypoxic response suggesting that they may play different roles determined by the environment.

The *Δilv3A* and *Δilv3A Δilv3B* strains appeared avirulent in a low dose mouse model of disseminated *A. fumigatus* infection (Figure 4A) indicating that AfIlv3A/IlvC function is required for virulence. The results of the pulmonary model further confirmed the requirement for AfIlv3A/IlvC however it is clear that the *Δilv3A* strain is not avirulent. Loss of both Ilv3A and Ilv3B impacted the pathogenicity of *A. fumigatus* to a greater level. This is likely to be a consequence of the combined effect of the loss of the two Ilv3 genes on branched chain-amino acid biosynthesis. We conclude therefore, that branched-chain amino acid biosynthesis, particularly with reference to AfIlv3A/IlvC and AfIlv3B, is an attractive pathway to target for antifungal drug development. In addition, the assay described in this manuscript is amenable to high-throughput screening of compounds to identify suitable inhibitors for a drug discovery project.

## Materials and Methods

### Virulence studies

For the low inoculum dissemination model, mice were rendered neutropenic with a single dose of 200 mg/kg intraperitoneally (IP) cyclophosphamide 3 days before infection to produce temporary neutropenia (until 2 days post infection). 2.5×10^5^ spores of the indicated strains were inoculated into the lateral tail vein of groups of 3–5 mice. For measurement of *A. fumigatus* tissue burden mice were euthanised 48 h, 72 h and 96 h post-infection and the burden of *A. fumigatus* in the kidneys was quantified by culture on Sabouraud dextrose agar (Scientific Laboratory Supplies). Burden was expressed as colony forming units per gram (cfu/g) of tissue. For survival studies, groups of 5 similarly immunosuppressed mice were inoculated with 2.5×10^5^ *A. fumigatus* spores and monitored for 7 days.

For the high inoculum inhalational model of invasive pulmonary aspergillosis, mice were rendered neutropenic on days minus 2 and plus 3 with 200 mg/kg IP cyclophosphamide, which resulted in profound and persistent neutropenia for 6 days. Cortisone acetate (Sigma) 250 mg/kg subcutaneously was also administered on days minus 2 and plus 3 to impair the function of pulmonary alveolar macrophages. A conidial suspension containing 1×10^9^ conidia/mL was prepared in phosphate buffered saline (Invitrogen) from 7-day old cultures on Sabouraud dextrose agar. Mice were exposed to 12 mL of this solution, which was nebulized (Hudson RCI, High Wycombe, UK) at 1 bar for 1 h. The experiment was continued 14 days post inoculation.

All experiments were performed under UK Home Office project license PPL40/3101 and approved by The University of Manchester Ethics Committee. Male CD1 mice (Charles River Ltd) were used (n = 6 per cage), weighing 22–25 g, and were stored in vented HEPA-filtered cages with free access to food and water.

### Strains and growth conditions

Strains used in this study are shown in Table 2. Strains were maintained at 37°C on Sabouraud dextrose agar (Oxoid) supplemented with 10 mM uridine,10 mM uracil, 1 mM isoleucine, 1 mM leucine and 1 mM valine where appropriate. Growth studies were carried out on minimal media [28] using 1 mM proline as a nitrogen source unless stated otherwise. For radial growth analysis 200 spores were spotted onto the centre of a minimal agar plate and incubated at 37°C for three days.

**Table 2:** *A. fumigatus* strains used in this study.

Strain	Genotype	Source
CEA10		FGSC
KU80ΔpyrG-	ak*uA^KU80^ pyrG-*	FGSC
*Δilv3A*	*ak*uA^KU80^ pyrG- *ilv3A(AFUA_2G14210)::pyrG*	this study
*Δilv3B*	ak*uA^KU80^ pyrG- ilv3B(AFUA_1G03550)::pyrG*	this study
*Δilv3A Δilv3B*	ak*uA^KU80^ pyrG- ilv3B*::*pyrG ilv3A::pyrG-*	this study
*Δilv3A::ilv3A*	ak*uA^KU80^ pyrG-*	this study
*Δilv3A Δilv3B::ilv3B*	ak*uA^KU80^ pyrG- ilv3A::pyrG-*	this study
*Δilv3A Δilv3B Rec*	ak*uA^KU80^ pyrG ilv3A::pyrG-*	this study
*Δilv3A Rec*	ak*uA^KU80^*	this study

### Phylogenetic analysis

ILV sequences were identified by BLAST searches at NCBI (http://blast.ncbi.nlm.nih.gov/Blast.cgi) and CADRE (http://www.cadre-genomes.org.uk/) [29] and from examination of the ILVD_EDD family entry of Pfam (http://pfam.sanger.ac.uk/) [30]. A full list of sequences used and their accession codes can be found in the legend to Figure 1. In the case of AfIlv3B (AFUA_1G03350), Anid2 (AACD01000066.1) and Ater4 (XP_001217288.1) it was necessary to improve the automated gene prediction.

Phylogenetic analysis was carried out using MrBayes [31], TREE-PUZZLE 5.2 [32], and PHYLIP [33], using the more conserved and unambiguously aligned regions of the ILV alignment. Bayesian inference of phylogeny was carried out using MrBayes with a gamma rates setting and a fixed (WAG) amino acid model. 300,000 generations were run with a sample frequency of 50 and a temperature value of 0.05. The first 170,000 generations were discarded prior to analysis. Maximum likelihood analysis was carried out using TREE-PUZZLE with the WAG model and 200,000 puzzling steps. Pairwise distances were calculated using PROTDIST with the JTT model for 100 bootstrap replicates generated using SEQBOOT; Trees were inferred using NEIGHBOR with jumbling of input order, and bootstrap values were calculated using CONSENSE. Trees were visualised using TREEVIEW [34].

### Determination of 5′ translation initiation site by RACE

To determine the 5′ ends of the genes, RACE (Rapid Amplification of cDNA Ends) was carried out, using the GeneRacer™ Kit (Invitrogen), as per manufacturers instructions. Briefly, dephosphorylated and de-capped *A. fumigatus* mRNA was ligated to the GeneRacer™ RNA Oligo. This preparation was used to prepare RACE-ready cDNA which was then used as a template in PCR using the Generacer sense primer (Invitrogen) and the gene specific antisense primer (RACE_ILV3A and RACE_ILV3B, see Table 3). RACE was carried out as follows: 94°C 2 min; (94°C, 30 sec; 72°C, 60 sec)×5; (94°C, 30 sec; 70°C, 60 sec)×5; (94°C, 30 sec; 64°C, 30 sec; 68°C, 60 sec)×30; 68°C, 600 sec.

**Table 3:** Primers used in this study.

Primer	Sequence (5′-3′)
***Gene Deletion***	
ILV3A_F1	GCCACAGCTGAACTTTCTCC
ILV3A_R1	ACGGTATTGACTAAAAGGGATCTAGGTAGCTGTATGGTTTCTAGATT
ILV3A_F2	TTCGAGCTCGGTACCCGGGGATCTTTACATGTGTGATAAGGGCGCAT
ILV3A_R2	CTTACGAACATGGCAACCGG
ILV3A_F3	AATGCGGCCGCAGTGTGCCCATCAAGCCACG
ILV3A_R3	AATGCGGCCGCCAGTGCATTCGGGACGAGTA
pyr_ILV3A_F	AATCTAGAAACCATACAGCTACCTAGATCCCTTTTAGTCAATACCGT
PYR_ILV3A_R	ATGCGCCCTTATCACACATGTAAAGATCCCCGGGTACCGAGCTCGAA
ILV3B_F1	CTTAATCTACGGGACTTGAC
ILV3B_R1	ACGGTATTGACTAAAAGGGATCTACATACAGAAACCAAGTATGATGTG
ILV3B_F2	TTCGAGCTCGGTACCCGGGGATCTGATCGACGAAAGTCCACTGTAAT
ILV3B_R2	ATGTCGATAGTCCGCACCCA
ILV3B_F3	AATGCGGCCGCGTACGATGAGCTCAACAAAC
ILV3B_R3	AATGCGGCCGCAACCAGCAATGGTATTGGTG
PYR_ILV3B_F	CACATCATACTTGGTTTCTGTATGTAGATCCCTTTTAGTCAATACCGT
PYR_ILV3B_R	ATTACAGTGGACTTTCGTCGATCAGATCCCCGGGTACCGAGCTCGAA
PYRG_F	TAAAATGCCAGTTCCGTCGT
PYRG_R	GCGTTTCTGGCAAAGCTTAC
***5′RACE***	
RACE_ILV3A	TGGGTTGTGTGACATTGCGGGAGAC
RACE_ILV3B	CAATGCATGCATCATGATACTGCGCGCAAGT
***Protein Expression***	
LIC_Ilv3A_F	GACGACGACAAGATGAAAGATAGCGAAGAAACGGCTT
LIC_Ilv3A_R	GAGGAGAAGCCCGGTTCATTCTACAGAGTCAGTGATGC
LIC_Ilv3B_F	GACGACGACAAGATGGACTCCTCTACCTCCGCGTCGTCCAA
LIC_Ilv3B_R	GAGGAGAAGCCCGGTCTAGAACAAATCCGT CATTGCACCATG

A ∼350 bp ILV3A PCR product was cloned into pGEM-T Easy (Promega) and a ∼550 bp ILV3B PCR product was cloned into pCR4-Topo (Invitrogen) and sequenced using T7 and T3 primers.

### Deletion of ilv3A and ilv3B genes in *Aspergillus fumigatus*

Approximately 2 kbp of 5′ and 3′ flanking regions of AfIlv3A and AfIlv3B were amplified by PCR from *A. fumigatus* genomic DNA using KOD DNA polymerase (Novagen). The primers ILV3A_F1 and ILV3A_R1 were used to amplify the 5′ and ILV3A_F2 and ILV3A_R2 were used to amplify the 3′ non-coding regions of the AfIlv3A gene (Table 3). The primers ILV3B_F1 and ILV3B_R1 were used to amplify the 5′ and ILV3B_F2 and ILV3B_R2 were used to amplify the 3′ non-coding regions of the AfIlv3B gene (Table 3). The orotidine 5′-monophosphate decarboxylase (*pyrG*) gene of *A. nidulans* was amplified incorporating ILV3 overhangs with PYR_ILV3A_F and PYR_ILV3A_R for preparation of the AfIlv3A deletion construct and PYR_ILV3B_F and PYR_ILV3B_R for preparation of the AfIlv3B deletion construct. The PCR products were purified (Qiaquick PCR purification, Qiagen) and deletion constructs prepared by fusion PCR. The three appropriate fragments were mixed and subjected to fusion PCR using ILV3A_F3 and ILV3A_R3 for the AfIlv3A deletion construct and ILV3B_F3 and ILV3B_R3 for the AfIlv3B deletion construct. Fusion PCR products were purified before being used to transform *A. fumigatus*.

*A. fumigatus* mycelia were treated with Glucanex (Novozymes) for 2–3 h at 30°C to produce protoplasts. DNA was transformed into protoplasts by PEG-mediated transformation [35]. Transformants were screened for homologous recombination by PCR and knockouts were confirmed by Southern blotting of genomic DNA.

Reconstituted isolates, i.e. Δilv3A::ilv3A and Δilv3A Δilv3B::ilv3B were generated by restoring the named genes back to their original locus using fragments amplified with ILV3A_F1/ILV3A_R2 and ILV3B_F1/ILV3B_R2 respectively. In both cases the reconstituted strains were selected on minimal media containing supplemental uridine, uracil and isoleucine. The *pyrG* gene was restored in the reconstituted strains by transforming with an *AfpyrG* gene fragment amplified with primers pyrG_F and pyrG_R.

### Preparation of recombinant AfIlv3A and AfIlv3B

A truncated version of the AfIlv3A coding sequence, excluding the N-terminal 29 amino acids that form the mitochondrial targeting sequence, was amplified from *A. fumigatus* cDNA by PCR using KOD DNA polymerase. The primers LIC_Ilv3A_F and LIC_Ilv3A_R (Table 3) that contain extensions that are homologous to vector were used. 5% DMSO was included to aid amplification of the GC-rich ILV3A coding sequence (61% GC). The PCR product was cloned into the pET30 Ek/LIC vector (Novagen) by ligation independent cloning following the manufacturers instructions. Sequencing confirmed that the resulting vector, pET30_Ilv3A, was correctly constructed.

pET30_Ilv3A was transformed into *E. coli BL21 DE3* (Novagen) and expression was induced by IPTG as directed in the manufacturers protocol. The culture was incubated for 20 h at 20°C prior to protein purification. Cells were lysed using BugBuster (Novagen) and protein purified by Immobilised Metal Affinity Chromatography (IMAC) using Ni-NTA His-Bind resin (Novagen). Buffer exchange was carried out on the eluate using a PD10 column (GE Healthcare) with the Ilv3B protein fraction being finally eluted in 62.5 mM Tris-HCl, 12 mM MgCl_2_ buffer, pH 8.0.

The AfIlv3B coding sequence was amplified from *A. fumigatus* cDNA using primers LIC_Ilv3B_F and LIC_Ilv3B_R (Table 3) using KOD DNA polymerase. The PCR product was cloned into the pET30 Ek/LIC vector to create pET30_Ilv3B. The Ilv3B protein was expressed in *E. coli BL21 DE3* as described above for AfIlv3A. However, resultant AfIlv3B was found in the insoluble fraction

### Assay for DHAD Activity

AfIlv3A protein (typically 2 µg per assay) was assayed in 50 mM Tris-HCl, 9.6 mM MgCl_2_, pH 8.0 based on a previously described methodology [23]. The reaction was started by the addition of the substrate L-threonic acid (Sigma-Aldrich). 10 mM L-threonic acid was used in standard assays, but variable amounts were added for Km determination. The reaction was allowed to proceed for 20 min at room temperature (22–24°C) before the reaction was stopped by the addition of 44 mM semicarbazide hydrochloride and 89 mM sodium acetate. The absorbance of the sample was read at 250 nm following a further 15 min incubation.

## Supporting Information

Figure S1 **Confirmation of gene disruption.** **A.** Southern blot analysis of *Avr*II (vertical arrows) digests of genomic DNA isolated from 1: CEA10; 2: *Δilv3B*; 3: *Δilv3A*; 4: *Δilv3A::ilv3A*. A probe directed against the *A. nidulans pyrG* (grey highlight) was used to show the presence of only a single copy of the knockout cassette in the Δilv3A strain and confirm the absence of the cassette in the Δilv3A::ilv3A strain. The locations of the primers that delimit the boundaries of the KO cassette are shown in the schematic (horizontal arrows). **B.** Southern blot analysis of *Bam*HI (vertical arrows) digests of genomic DNA isolated from 1: CEA10; 2: *Δilv3A*; 3: *Δilv3B*; 4: *Δilv3AΔilv3B* and 5: *Δilv3AΔilv3B::ilv3B*. A probe directed against the 5′ flank of the ilv3B gene (grey highlight) was used to show correct integration of the knockout cassette in the Δilv3B *Δilv3AΔilv3B* and strains and confirm the reincorporation of Δilv3B to the wild-type locus in the *Δilv3AΔilv3B::ilv3B* strain. Absence of additional hybridizing bands confirms the integration of the cassettes as single copies. The locations of the primers that delimit the boundaries of the KO cassette are shown in the schematic (horizontal arrows). Low level none specific hybridization of the probe can be seen in 1, 2 and 5. This is masked in 3 and 4 however this does not affect the interpretation of the blot.
(TIF)
