# Supplementary figures and images for: The Aspergillus fumigatus Dihydroxyacid Dehydratase Ilv3A/IlvC Is Required for Full Virulence
Source: PLoS One. 2012 Sep 18;7(9):e43559. doi: 10.1371/journal.pone.0043559 (PMC3445565; doi:10.1371/journal.pone.0043559)

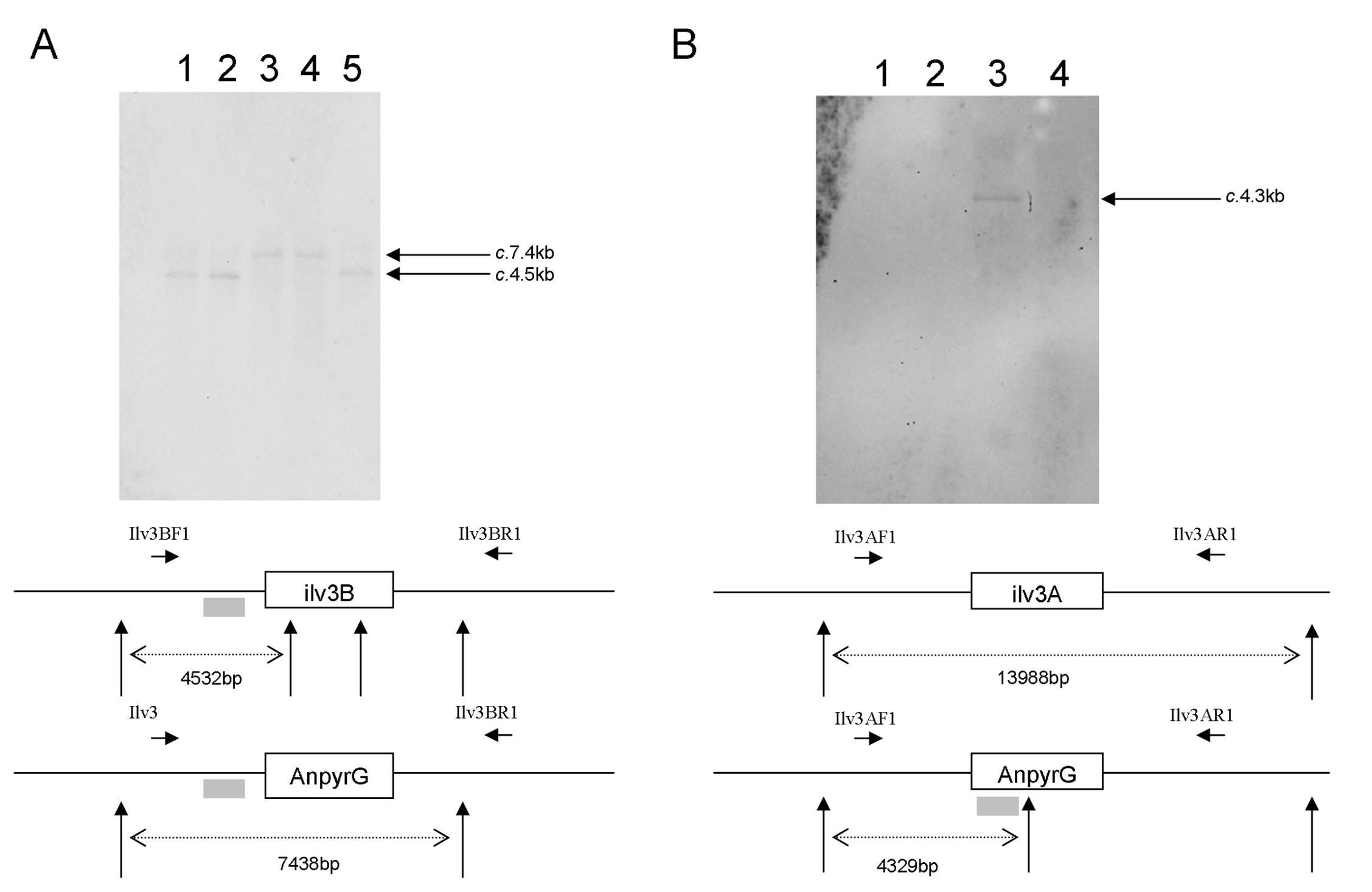

Supplement: Figure S1 — Confirmation of gene disruption. A. Southern blot analysis of AvrII (vertical arrows) digests of genomic DNA isolated from 1: CEA10; 2: Δilv3B; 3: Δilv3A; 4: Δilv3A::ilv3A. A probe directed against the A. nidulans pyrG (grey highlight) was used to show the presence of only a single copy of the knockout cassette in the Δilv3A strain and confirm the absence of the cassette in the Δilv3A::ilv3A strain. The locations of the primers that delimit the boundaries of the KO cassette are shown in the schematic (horizontal arrows). B. Southern blot analysis of BamHI (vertical arrows) digests of genomic DNA isolated from 1: CEA10; 2: Δilv3A; 3: Δilv3B; 4: Δilv3AΔilv3B and 5: Δilv3AΔilv3B::ilv3B. A probe directed against the 5′ flank of the ilv3B gene (grey highlight) was used to show correct integration of the knockout cassette in the Δilv3B Δilv3AΔilv3B and strains and confirm the reincorporation of Δilv3B to the wild-type locus in the Δilv3AΔilv3B::ilv3B strain. Absence of additional hybridizing bands confirms the integration of the cassettes as single copies. The locations of the primers that delimit the boundaries of the KO cassette are shown in the schematic (horizontal arrows). Low level none specific hybridization of the probe can be seen in 1, 2 and 5. This is masked in 3 and 4 however this does not affect the interpretation of the blot. (TIF) [file pone.0043559.s001.tif]
